# Genome wide analysis of carotenoid cleavage oxygenases (*CCO*) gene family in *Arachis hypogaea* (peanut) under biotic stress

**DOI:** 10.1186/s12864-024-10351-w

**Published:** 2024-05-14

**Authors:** Arsalan Ahmad, Adnan Sami, Umer Habib, Muhammad Ali, Muhammad Shafiq, Muhammad Zeshan Haider, Shahbaz Ahmad, Baber Ali, Steve Harakeh, Rania M. Makki, Talha Chaudhary, Fathia A. Soudy

**Affiliations:** 1 Department of Entomology, Faculty of Agricultural Sciences, University of the Punjab, Lahore, 54590 Pakistan; 2 Department of Plant Breeding and Genetics, Faculty of Agricultural Sciences, University of the Punjab, Lahore, 54590 Pakistan; 3 Department of Horticulture, PMAS Arid Agriculture University, Rawalpindi, 46000 Pakistan; 4 Department of Entomology, Faculty of Agricultural Sciences, University of the Punjab, Lahore, 54590 Pakistan; 5 Department of Horticulture, Faculty of Agriculture Sciences, University of the Punjab, Lahore, 54590 Pakistan; 6 Department of Plant Breeding and Genetics, Faculty of Agricultural Sciences, University of the Punjab, Lahore, 54590 Pakistan; 7 Department of Entomology, Faculty of Agricultural Sciences, University of the Punjab, Lahore, 54590 Pakistan; 8 Department of Plant Sciences, Quaid-i-Azam University, Islamabad, 45320 Pakistan; 9 King Fahd Medical Research Centre, King Abdulaziz University, Jeddah, Saudi Arabia; 10 Yousef Abdul Latif Jameel Scientific Chair of Prophetic Medicine Application, Faculty of Medicine, King Abdulaziz University, Jeddah, Saudi Arabia; 11 Department of Biological Sciences, Faculty of Science, King Abdulaziz University (KAU), Jeddah, 21589 Saudi Arabia; 12 Faculty of Agricultural and Environmental Sciences, Hungarian University of Agriculture and Life Sciences, Godollo, 2100 Hungary; 13 Genetics and Genetic Engineering Department, Faculty of Agriculture, Benha University, Moshtohor, 13736 Egypt

**Keywords:** GWAS, Genomics, Carotenoids, Peanut, Oxygenases, Enzyme

## Abstract

Carotenoid cleavage oxygenases (*CCOs*) enzymes play a vital role in plant growth and development through the synthesis of apocarotenoids and their derivative. These chemicals are necessary for flower and fruit coloration, as well as the manufacture of plant hormones such as abscisic acid (ABA) and strigolactones, which control a variety of physiological processes. The *CCOs* gene family has not been characterized in *Arachis hypogaea*. Genome mining of *A. hypogaea* identifies 24 *AhCCO* gene members. The *AhCCO* gene family was divided into two subgroups based on the recent study of the Arabidopsis thaliana *CCO* gene family classification system. Twenty-three *AhCCO* genes, constituting 95.8% of the total, were regulated by 29 miRNAs, underscoring the significance of microRNAs (miRNAs) in governing gene expression in peanuts. *AhCCD19* is the only gene that lacks a miRNA target site. The physicochemical characteristics of *CCO* genes and their molecular weights and isoelectric points were studied further. The genes were then characterized regarding chromosomal distribution, structure, and promoter cis-elements. Light, stress development, drought stress, and hormone responsiveness were discovered to be associated with *AhCCO* genes, which can be utilized in developing more resilient crops. The investigation also showed the cellular location of the encoded proteins and discovered that the peanut carotenoid oxygenase gene family’s expansion was most likely the result of tandem, segmental, and whole-genome duplication events. The localization expresses the abundance of genes mostly in the cytoplasm and chloroplast. Expression analysis shows that *AhCCD7* and *AhCCD14* genes show the maximum expression in the apical meristem, lateral leaf, and pentafoliate leaf development, while *AhNCED9* and *AhNCED13* express in response to Aspergillus flavus resistance. This knowledge throws light on the evolutionary history of the *AhCCO* gene family and may help researchers better understand the molecular processes behind gene duplication occurrences in plants. An integrated synteny study was used to find orthologous carotenoid oxygenase genes in *A. hypogaea*, whereas *Arabidopsis thaliana* and Beta vulgaris were used as references for the functional characterization of peanut *CCO* genes. These studies provide a foundation for future research on the regulation and functions of this gene family. This information provides valuable insights into the genetic regulation of *AhCCO* genes. This technology could create molecular markers for breeding programs to develop new peanut lines.

## Introduction

 Carotenoid cleavage oxygenase (*CCO*) is an enzyme that plays a role in the biosynthesis of the phytohormones ABA and strigolactone (SL) as well as in biological functions such as light absorption, electron transfer, and oxygen-free radical removal [1]. These are non-heme iron oxygenases that help in carotenoids oxidate remodeling and produce apocarotenoids, which control phytohormone regulation, pigments, and defensive compounds in plants [2]. There are approximately 50 carotenoids that are specifically precursors of vitamin A, which play a key role against cancer disease. It also contributes to the color of fruits like red, yellow, and orange [3]. *CCO* have has conserved histidine that might help in iron-binding and also contain a peptide sequence at the carboxyl terminus that constitutes a family signature sequence. In *A. thaliana*, the *CCO* gene family have nine members [4]. Out of nine, two *CCDs* are involved in sequential cleavage that is stimulated by *CCD7* and *CCD8*, which produce shoot-branching inhibiting hormone [5]. The *CCO* genes belong to a long-established family. The maize (*Zea mays*) abscisic acid -deficient viviparous mutant, vp14, had the first *CCO* gene expression. Later, nine vp14 homologs were found in *A. thaliana* [6]. These are terpenoid substances made by phototrophic organisms, such as plants, certain eubacteria, and fungi [7]. In order to create different apocarotenoids and their derivatives, *CCOs* can precisely cleave the conjugated double bond of the carotenoid polyene chain. Plant *CCOs* can be further divided into the 9-cis-epoxycarotenoid (*NCED*) and carotenoid cleavage dioxygenase (*CCD*) subfamilies based on whether the substrates are epoxidized.

Abscisic acid (ABA) is a 15-C weak acid discovered in the 1960s that has a variety of functions in plant development and growth, including embryo maturation, seed dormancy, germination, cell division and elongation, floral induction, and responses to environmental challenges such as pathogen attack, dehydration, salinity etc [8]. Initially thought to be a growth inhibitor, abscisic acid is now recognized as a crucial plant hormone that controls plant growth [9]. ABA not only regulates the stomatal pathway but is also involved in gene expression and regulating cis-acting elements [10]. Disease resistance is negatively regulated by ABA’s interference with biotic stress signaling, which is controlled by salicylic acid, jasmonic acid, and ethylene [11].

In plants, carotenoid cleavage dioxygenase (*CCD*) emits several volatile phytohormones and aroma compounds. The actual cleavage mechanism is still not understood. This enzyme group has more importance in terms of nutritional value. These hormones cause symbiotic mycorrhizal fungi to branch and attach to plant roots, or they can be transmitted to plant aerial portions to prevent branching or tillering, which is a major agronomic feature in grasses [12]. *CCD* is known specifically for its double bond cleavage, which is different in plants and animals. The catalization occurs at 9, 10, and 9′, 10′ double bonds [13]. Carotenoids serve as precursors in the synthesis of regulatory molecules within plants, including β-cyclocitral, as well as newly identified regulatory metabolites like anchorene, iso-anchorene, and zaxinone. These derivatives are made by selective oxidative cleavage of carotenoids, primarily triggered by *CCDs* [14]. *CCD* genes have distinct functions in different crops: in tomatoes, they contribute to flavor volatiles; in petunias, β-ionone (a recognized key contributor to floral fragrance in various plant species [15]) synthesis; and in *Brassica oleracea*, responses to drought and salt stresses [2]. In grapevine, *CCD* is known for producing the flavor and aroma related apocarotenoid in both fruits and flowers [5]. In the grapevine, it has been discovered that a total of nine *CCDs* genes play a crucial role in the synthesis of the shoot-branching inhibiting hormone, strigolactone. This synthesis process is specifically enhanced through sequential cleavage catalyzed by the *Arabidopsis CCD7* and *CCD8* genes [16].

9-cis-epoxycarotenoid dioxygenase (*NCED*) is also an essential enzyme that stimulates ABA biosynthesis, as shown by genomic studies of plants [17]. It was cloned from the maize plant and then from other plant species responsible for the production of xanthoxin (an intermediate compound in ABA synthesis) [18]. Now the *NCED* gene has been translated and expressed in various plants, including beans, cowpeas, avocados, grapes, *Arabidopsis*, sweet cherries, and citrus, since it was first discovered in the maize vp14 mutant. In sweet cherries, the *NCED* gene (*PavNCED5*) influences the bud dormancy [19]. The expression of *NCED (PvNCED1*) in *Phaseolus vulgaris* under drought conditions demonstrated a substantial connection between *NCED (PvNCED1*) and ABA levels in dry leaves and roots, suggesting that *NCED* controls ABA production under water stress [20]. Tobacco and tomato were the first plants in which overexpression of *NCED* resulted in excessive accumulation of ABA. In tomato, this led to greater water usage efficiency due to an increase in root hydraulic conductivity and a decrease in stomatal conductance (g_s_) [21]. In rice, the *NCED* genes *OsNCED6* and *OsNCED10* defend against salt stress, whereas *OsCCD7* and *OcCCD8* are involved in grain and inflorescence development [22].The genes *HaNCED16* and *HaNCED19* were expressed in response to drought stress revealed in recent studies [23].

Peanuts are an economical and nutritious source of food worldwide. *A. hypogaea* produces different products like peanut butter and peanut oil that help to meet nutritional values as well as lower body mass index (BMI) [24]. A total of 24 *AhCCO* genes were discovered that might play a role in peanuts’ resistance to various fungal pathogens. The cis-elements of *A. hypogaea* regulate responses to low temperature and light, anaerobic induction, jasmonate signaling, and both biotic and abiotic stress [25, 26]. ABA plays a key role in regulating physiological processes like fruit development, biotic and abiotic stresses. In this study, we identified *CCO* genes from the peanut genomes and investigated their evolutionary relationship, gene structure, protein motifs, and expression profiles in various organs, stages of fruit development, and responses to abiotic and biotic stress. This discovery expands our understanding of *CCO* genes involved in growth, development, and stress responses and provides the basis for peanut genetic enhancement.

## Materials and methods

### Identification of the *CCO*gene family, Physiochemical properties and subcellular localization

The *CCO* protein sequence was retrieved from the National Center for Biotechnology Information (NCBI) with the domain RPE65. The protein sequence of *A. hypogaea*, along with *(A) thaliana* and *(B) vulgaris*, was retrieved from the phytozome database (https://phytozome-next.jgi.doe.gov/, accessed on June 2, 2023). The genes with conserved RPE65or PF03055 domains were considered the true *CCO* genes. Physicochemical properties of the *CCO* protein in *A. hypogaea*, such as mRNA length, chromosomal start and end position taken from the Phytozome and other parameters such as number of amino acids, molecular weight, theoretical pI value, grand average of hydropathicity (GRAVY), instability index, were taken from the ExPASy (https://web.expasy.org/protparam/, accessed on June 4th, 2023) online tool and protein subcellular localization prediction using WoLF PSORT (https://wolfpsort.hgc.jp/, accessed on June 7th, 2023).

### Analysis for comparative phylogenetic tree

The analysis was performed between the peptide sequences of *A. hypogaea*, *(A) thaliana*, and *(B) vulgaris* for the construction of a phylogenetic tree. The *CCO* sequence of proteins that was used to make a phylogenetic tree in MEGA-11 (https://www.megasoftware.net/) software with the method of neighbor-joining (NJ) and setting 1000 replications for the bootstrapping value. The final structure was done on iTOL (https://itol.embl.de/).

### Conserved motif analysis and gene structure

The conserved motifs of *CCO* gene family proteins in *A. hypogaea* were examined using Multiple Expectation Maximization for Motif Elicitation (MEME) (https://meme-suite.org/meme/tools/meme). The motif’s maximum value was set to 20, and its maximum length ranged from 6 to 50. The conserved motifs were then shown using the TB-tools (https://bio.tools/tbtools) program. The intron exon substructure of *CCO* family genes was visualized using analytical tools from the Gene Structure Display Server (GSDS, https://gsds.gao-lab.org/ , accessed on June 13th, 2023).

### Chromosomal Location of *AhCCO *genes

Phytozome (https://phytozome-next.jgi.doe.gov/) provided details about the *CCO* locations, including the length of the chromosome, the chromosome it is on, and its precise location within the chromosome. Using the TB-tools software, the image displaying the location of *CCO* on the chromosome was created.

### Cis regulating gene analysis

The 1000-bp upstream sequence of the translation initiation codon of *AhCCO* was extracted from the Phytozome on June 15th, 2023.

PlantCare (https://bioinformatics.psb.ugent.be/webtools/plantcare/html/ , accessed on June 14th, 2023) database was used for cis-acting regulatory element prediction.

### MiRNA analysis and protein-protein interaction

Using the CDS sequences of all the *CCO* genes with the default parameters, psRNATarget (https://www.zhaolab.org/psRNATarget/ , accessed on June 17th, 2023) was used to find the *AhCCO* gene-related micro-RNA (miRNA) sequences. The STRING database was used to predict potential interactions among the proteins (https://string-db.org/ , accessed on June 18th, 2023).

### Gene duplication and synteny analysis

Ka/Ks ratios were used to estimate the divergence time of the *AhCCO* genes. The substitution rates were calculated using TB-tools. The Ks value was entered into the “T = Ks/2r” equation, where “r” is equal to 8.12*10^-9, to estimate the time of divergence (DT) [27]. MCScanX v1.0 (Multiple Collinearity Scan toolkit) was used to analyse the gene duplication occurrences. Syntenic and dual syntenic maps were created in order to determine the synteny link between the genes of *A. hypogaea*, *(A) thaliana* and *(B) vulgaris*.

### Transcriptomic analysis/gene expression analysis

RNA seq analysis data of the *AhCCO* gene was extracted (GEO; accessed on June 23rd, 2023; accession no. GSE180915) from different plant parts like the lateral leaflet and terminal leaflet using petioles and seeds to study the transcriptomic effect on compound leaf development in *A. hypogaea.* Another piece of data from the RNA-seq study of the *AhCCO* gene was used to study the resistance of peanut pre-harvest seeds in response to *Aspergillus flavus*. Data related to compound leaf development of peanut and response to *A. flavus* retrieved from the NCBI Gene Expression Omnibus (GEO) (accessed on June 26th, 2023; accession no. GSE102782).

## Results

### Identification of genes in peanut

In a genome-wide study of peanuts, 24 *CCO* genes were identified (Table 1). Gene names, chromosomal locations, chromosomal direction, gene lengths, exon numbers, molecular weights (Mw in kDa), protein size, and isoelectric points (pI) are also shown in Table 1. This study shows the coding sequence (CDS) ranges from 231 bp (bp) (*AhCCD4*) to max. 1905 bp (*AhCCD17*), while the molecular weight (Mw) lies between 8612.91 (*AhCCD4*) and 71589.99 (*AhCCD17*) in all 24 genes of peanut. The following *CCO* gene lengths range from 76 (*AhCCD4*) to 633 (*AhNCED9*), and pI values vary from 4.62 (*AhCCD19*) to 8.64 (*AhCCD3*).

**Table 1:** NCED and CCD gene in CCO gene family information for 24 non-redundant genes discovered in the peanut genome

Name	Phytozome ID	Chromosome number	Location (Base pairs)	Direction	mRNA (CDS)	Peptides	pl	Mw (kDa)	Exon	Gene Length
*AhCCD1*	arahy.Tifrunner.gnm1.ann1.B2GMFE	19	145987927.145988308	R	276	91	5.21	10246.70	2	381
*AhCCD2*	arahy.Tifrunner.gnm1.ann1.V9TV9N	19	145862955.145863357	F	276	91	5.18	10227.65	2	402
*AhCCD3*	arahy.Tifrunner.gnm1.ann1.U7NLJQ	19	146028279.146028684	R	267	88	8.64	9863.70	2	405
*AhCCD4*	arahy.Tifrunner.gnm1.ann1.U7I4L9	19	23074704.23075050	R	231	76	5.62	8612.91	3	346
*AhCCD5*	arahy.Tifrunner.gnm1.ann1.CEV0UN	19	145813346.145813748	F	276	91	5.21	10246.70	2	402
*AhCCD6*	arahy.Tifrunner.gnm1.ann1.X3TKXF	19	145935331.145935730	R	282	93	5.01	10387.87	2	399
*AhCCD7*	arahy.Tifrunner.gnm1.ann1.CX54DC	18	124694543.124704470	F	1656	551	6.19	62177.30	13	9927
*AhCCD8*	arahy.Tifrunner.gnm1.ann1.5NX4LW	18	115196503.115203144	F	768	255	6.80	28721.03	8	6641
*AhNCED9*	arahy.Tifrunner.gnm1.ann1.2M7K4U	13	143893473.143895375	F	1902	633	6.88	70169.58	1	1902
*AhCCD10*	arahy.Tifrunner.gnm1.ann1.66FUHL	10	100158463.100168446	F	1679	390	5.12	44933.10	9	9983
*AhCCD11*	arahy.Tifrunner.gnm1.ann1.MD1Q75	10	99462768.99468250	R	1689	562	6.99	62733.27	6	5482
*AhCCD12*	arahy.Tifrunner.gnm1.ann1.I05T7U	10	100100280.100107039	F	1356	451	8.27	51496.91	10	6759
*AhNCED13*	arahy.Tifrunner.gnm1.ann1.39HIQW	5	3182703.3185673	R	1779	592	6.25	64913.20	2	2970
*AhCCD14*	arahy.Tifrunner.gnm1.ann1.GV0PL5	8	44467850.44476410	F	1656	551	6.11	62203.24	14	8560
*AhCCD15*	arahy.Tifrunner.gnm1.ann1.8D7DUE	8	41156080.41163062	F	1617	538	5.79	60622.35	14	6982
*AhNCED16*	arahy.Tifrunner.gnm1.ann1.H0876V	15	3182703.3185673	R	1779	592	6.25	64913.20	2	2970
*AhCCD17*	arahy.Tifrunner.gnm1.ann1.6Q8RNL	6	106617605.106626111	R	1905	634	7.66	71589.99	7	8506
*AhNCED18*	arahy.Tifrunner.gnm1.ann1.8YR14Z	3	140917614.140919534	F	1920	639	6.39	71027.36	1	1920
*AhCCD19*	arahy.Tifrunner.gnm1.ann1.2N484J	14	94556303.94556622	F	234	77	4.62	8733.90	2	319
*AhCCD20*	arahy.Tifrunner.gnm1.ann1.35913I	14	10249518.10249861	R	261	86	4.74	9879.18	2	343
*AhCCD21*	arahy.Tifrunner.gnm1.ann1.YWII14	20	123096984.123107035	F	1440	479	7.62	54700.50	10	10,051
*AhCCD22*	arahy.Tifrunner.gnm1.ann1.I9LXD5	20	123155779.123175747	F	1659	552	5.55	62295.95	13	19,968
*AhCCD23*	arahy.Tifrunner.gnm1.ann1.4KPL6Q	20	122634148.122639825	R	1680	559	7.59	62206.61	6	5677
*AhCCD24*	arahy.Tifrunner.gnm1.ann1.CQPK1H	16	139563356.139571622	R	1851	616	7.30	69545.46	7	8266

*Abbreviation***:** *Mw* Molecular weight, *pI* Isoelectric point

### Analysis for miRNA (MicroRNA) target site

MiRNA target site prediction shows that there were 29 miRNA target sites in 23 (95.8%) *AhCCO* genes (including *CCD* and *NCED*). 12 mature miRNAs (nucleotides) specifically target the gene *AhCCD15*, whereas 11 miRNAs target the genes *AhNCED9* and *AhCCD22*. Other genes are targeted by mature miRNAs ranging from 1 to 10. *AhCCD19* is the only gene that lacks a particular miRNA. In all genes, the length of these miRNAs varies from 16 to 22. 20 miRNAs observed play a significant role in cleavage, while 25 control translation. Some of them are involved in both cleavage and translation.

### Conserved motif domain analysis for CCO gene family

**Fig. 1:**
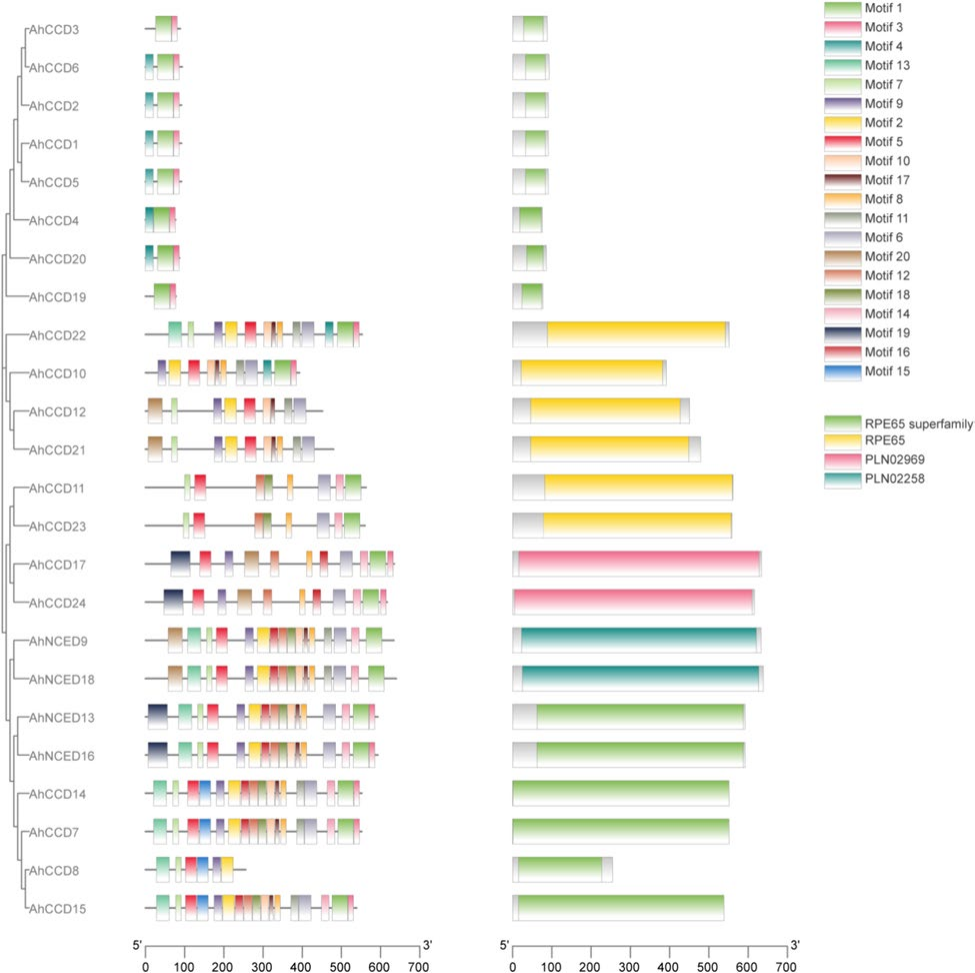
The distribution of 20 motifs of the peanut *CCO* protein family. Some member of the *NCED* and *CCD* protein has a conserved motif

The conserve domain motif analysis depicts that all motifs are conserved under the domain of the superfamily RPE65. In addition, Fig. 1 comprehensively shows that genes *AhCCD12, AhCCD19, *and* AhCCD20* have two motifs, while *AhCCD10, AhCCD21, AhCCD4, AhCCD3, AhCCD1, AhCCD2, AhCCD5*, and *AhCCD6* have 3 motifs only. All remaining genes have motifs that vary from six to seventeen, but motif-1 was conserved and is present in all genes of *A. hypogaea*, as shown in Fig. 1.

### Phylogenetic relationship of *CCO* protein

**Fig. 2:**
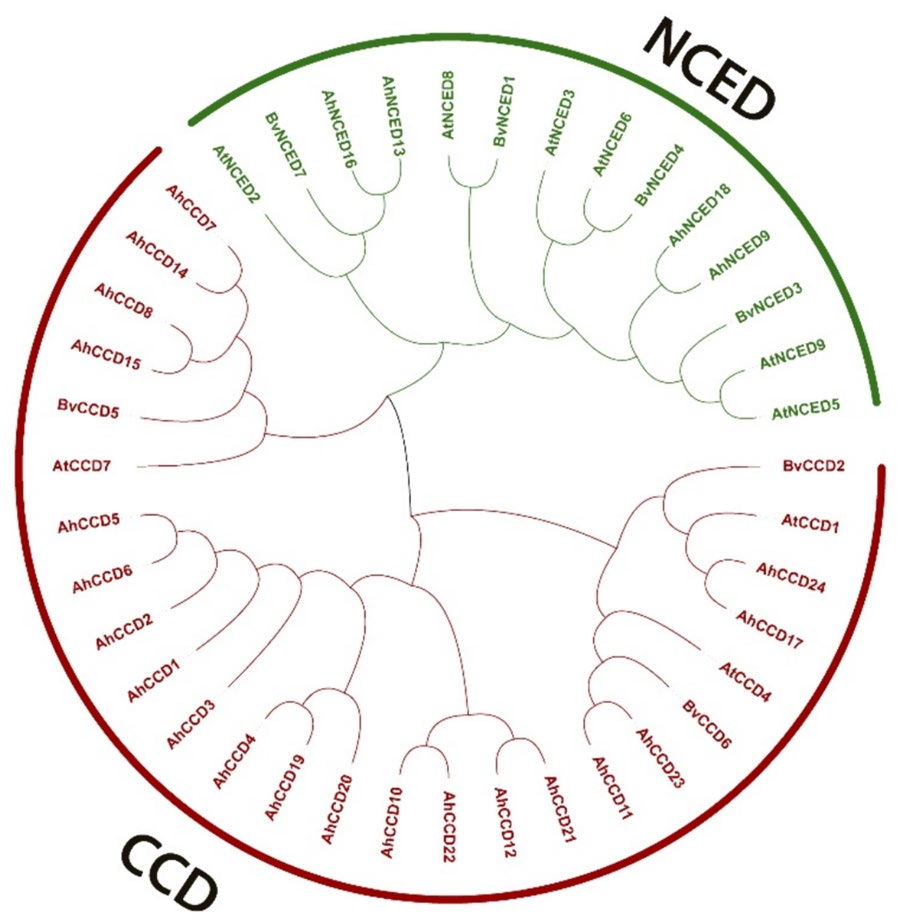
Illustrates the phylogenetic relationships among 40 *CCO* genes from three plant families: *A. hypogaea*, *(A) thaliana* and *(B) vulgaris*. Groupings are established through *Arabidopsis* phylogenetic tree reconstruction and evolutionary trait analysis. This figure visually represents the division of these 40 *CCO* genes among the three crop species, following the *Arabidopsis* phylogenetic grouping system

The construction of the phylogenetic tree of the *CCO* proteins of *A. hypogaea*, *B. vulgaris*, and *A. thaliana* was done in Mega-11 software (Fig. 2). The analysis exhibits 24 genes of *A. hypogaea*, seven of *B. vulgaris*, and nine of *A. thaliana* contributing to the phylogenetic tree. Genomic tree visualizes two main groups, *CCD* and *NCED*, in sugarbeet, peanut, and *Arabidopsis *evolutionary analysis*. *Group-1 contain all *CCD* genes shown in red, while Group-2 contain *NCED *shown in green.

### Cis regulatory element (CREs) analysis

**Fig. 3:**
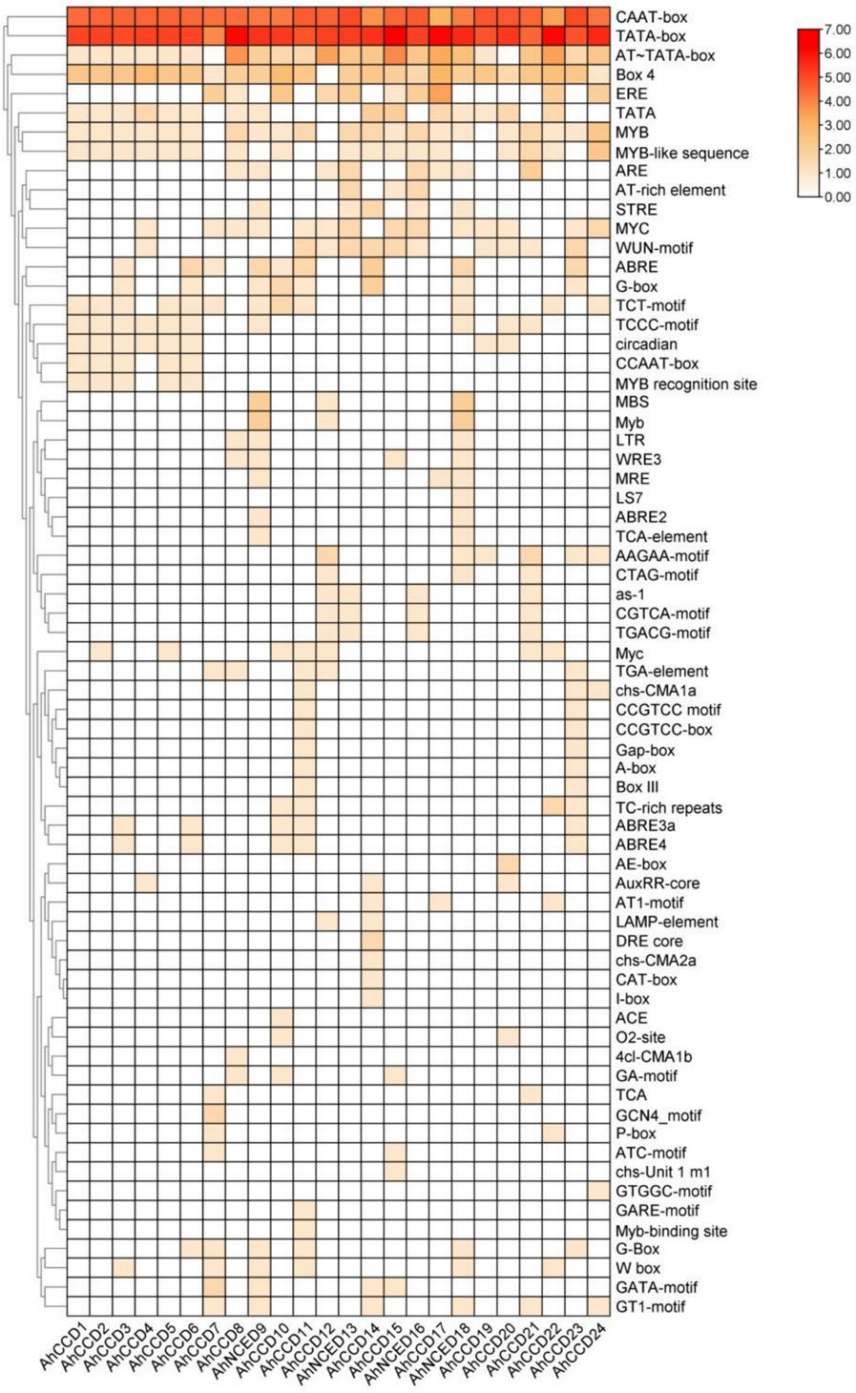
The graphical representation of cis-regulatory analysis of *CCO* genes with their function intensity. The intensity is defined by the range of red (highest) to white (lowest) during plant biochemical and physiological processes

The CAT-box, which is a cis-acting regulatory element related to meristem expression, was found to exist in one *AhCCO *promoter (Fig. 3). A number of cis-acting elements involved in phytohormone-responsiveness, such as AuxRR-core, CGTCA-motif, TGACG-motif, TGA-element, ABRE, P-box, GARE-motif, and TCA-element, were identified in the *CCO *promoters. Methyl jasmonate-responsive elements, including CGTCA-motif and TGACG-motif, appeared in most *CCO *promoters. Notably, ABRE, which is involved in ABA responsiveness, was observed in most of *CCO *promoters. There were also many types of stress-induced cis-acting elements in the *CCO *promoters, such as ARE, LTR, MBS, TC-rich repeats, DRE, and GC-motif. ARE is essential for anaerobic induction and was found in most promoters (seven out of 24 *AhCCO *promoter). TC-rich repeats, which are involved in defense and stress responsiveness, were detected in five *AhCCO *promoters. The LTR cis-acting elements, which are involved in low-temperature, responsiveness, were found in many promoters (three *AhCCO*). The DRE cis-acting element, which is involved in dehydration, low-temperature and salt stress, was specific to the *AhCCO *promoter, while the GC-motif involved in anoxic specific inducibility only existed in the *AhCCO *promoters. GTGGC-motif, I-box, LS7, chs-CMA2a, chs-Unit 1 m1, 4cl-CMA1b, and ACE that are present in genes *AhCCD24, AhCCD14, AhNCED18, AhCCD14, AhCCD15, AhCCD8, AhCCD10 *respectively play important role in regulating light-responsive elements, and GARE-motif controls the gibberellin-responsive element that play a role in signal transduction, grain germination, and certain transcription factors that regulate gene expression.

### Gene duplication and gene mapping of CCO genes

**Fig. 4:**
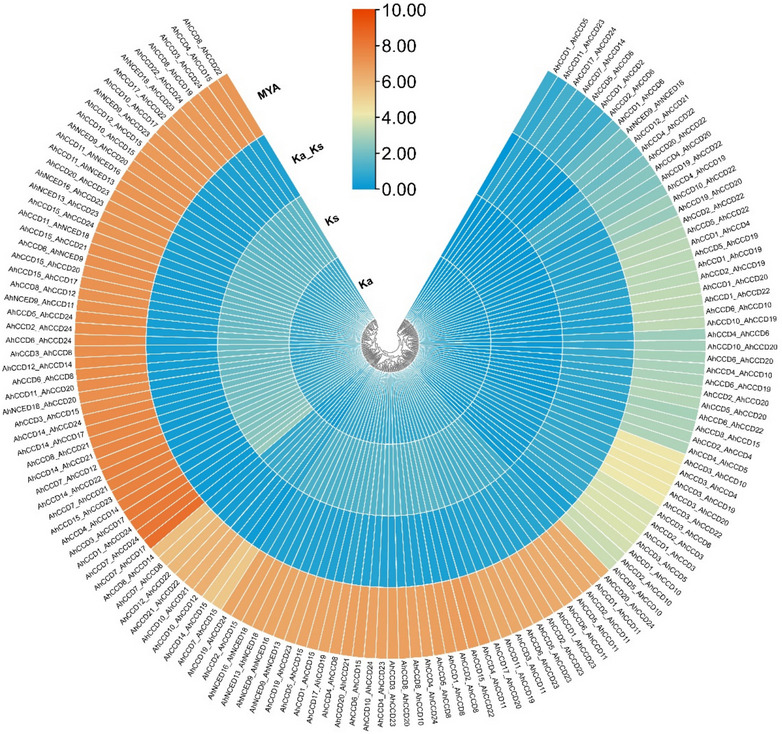
The ratio of mutations involving non-synonymous substitutions (Ka) to mutations involving synonymous substitutions (Ks) is shown as Ka/Ks

The gene duplication of *CCO *was calculated using TB-tools (Fig. 4). The ka/ks (non-synonymous substitution rate and synonymous substitution rate) values that show the *CCO* genes of *A. hypogaea *range from 0 (*AhCCD1_AhCCD5*), which is the lowest, to value 1.45 (*AhCCD20_AhCCD22*), which is the highest among all. The paralogous-gene pair *AhCCD7_AhCCD17* has the greatest value of 346.442478 (million years ago) and the lowest value of 0.786267402 (million years ago) of gene pair* AhCCD11_AhCCD23 *for segmental duplication. The ka/ks value less than 1 indicates purifying selection, except gene pairs (*AhCCD4_AhCCD20, AhCCD19_AhCCD22, AhCCD4_AhCCD22, and AhCCD20_AhCCD22*) have a value greater than 1 indicating positive or diversifying selection [28, 29]. Gene mapping analysis reveals the presence of a single gene on chromosomes 3, 5, 6, 13, 15, and 16. Chromosomes 8, 14, and 18 each harbor two genes, while chromosomes 10 and 20 contain three genes. Chromosome 19 stands out with six identified genes.

### Sub-cellular localization of *CCO *genes

**Fig. 5:**
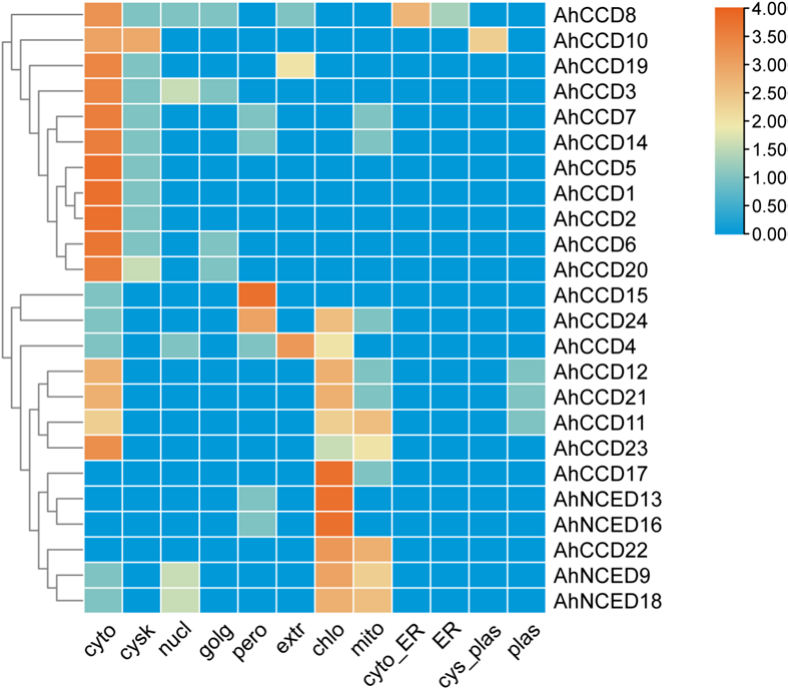
The heat map displays the sub-cellular distribution of *AhCCO* genes in plant cells, indicating their presence in the nucleus, cytoplasm, chloroplast, golgi apparatus, mitochondria, plasmid, and peroxisomes. Blue signifies absence, white suggests minimal functional presence, and mild orange indicates significant functional importance of the gene in the specific region

In peanuts, the localization of *CCO* genes within cells is abundant, most in the cytoplasm and chloroplast (Fig. 5). Other organelles, like the peroxisome and mitochondria, also contain *CCO* genes in quantity but less in number.

### Intron/exon structure analysis

**Fig. 6:**
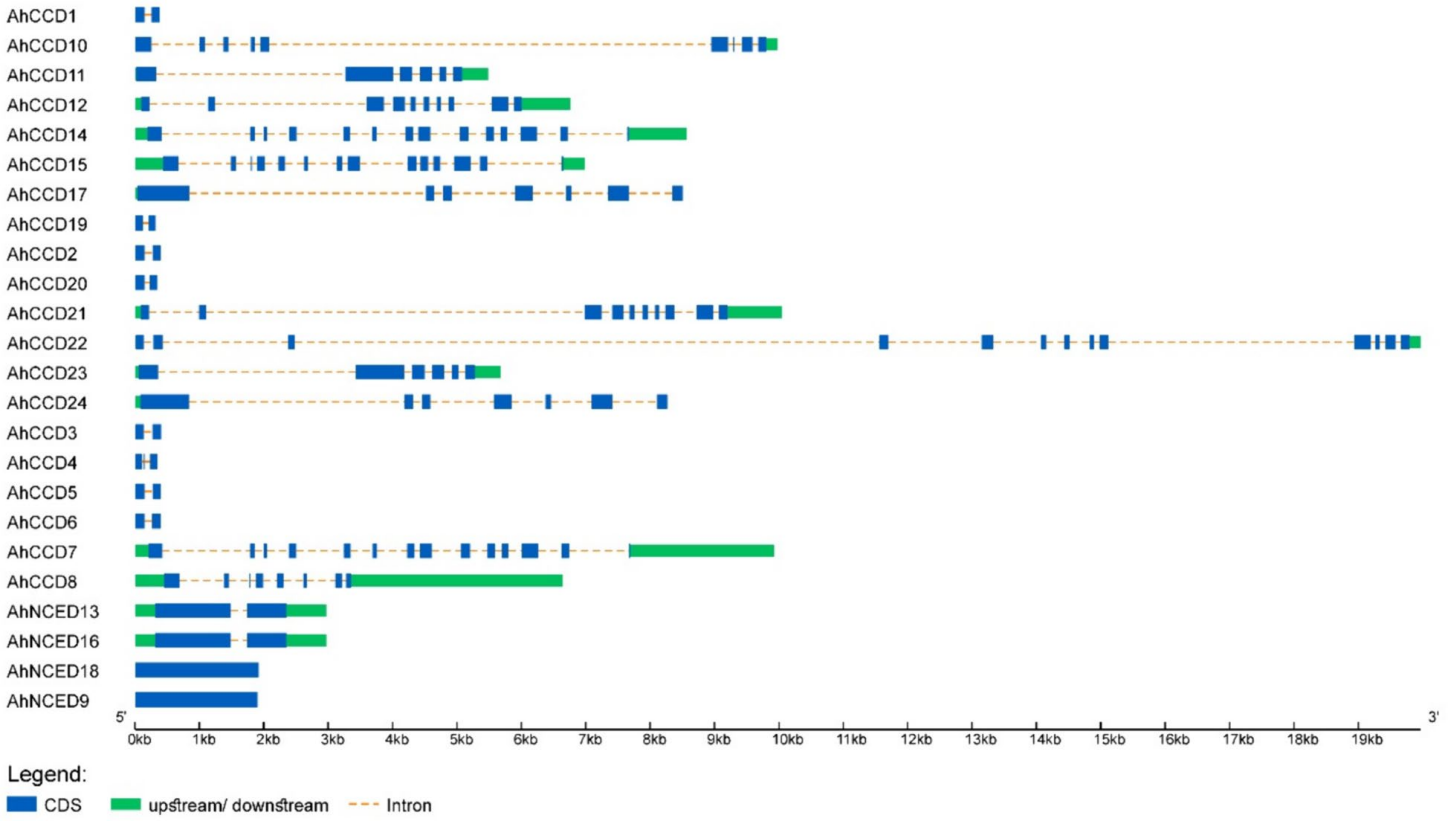
The phylogenetic representation of the intron-exon structure demonstrates that *NCED* genes have fewer coding sequences than *CCD* genes. In the meanwhile, the some *NCED* and *CCD* gene are conserved in the number of introns and exons. Genes *AhCCD14* and *AhCCD15* have highest exons of 14 while *AhNCED9* and *AhNCED18* have least of only one exon

The intron-exon analysis shows that 10 out of 24 genes have just two exons and one intron. These genes might be descended from a common ancestor. Two genes contain only one exon and no intron. Other genes have exon-intron ranges from 14–13 as the maximum to a 7–6 ratio as the minimum (Fig. 6). The research demonstrates how different genes with the same number of exons and introns predict gene structure.

### Protein-protein interaction of different genes

**Fig. 7:**
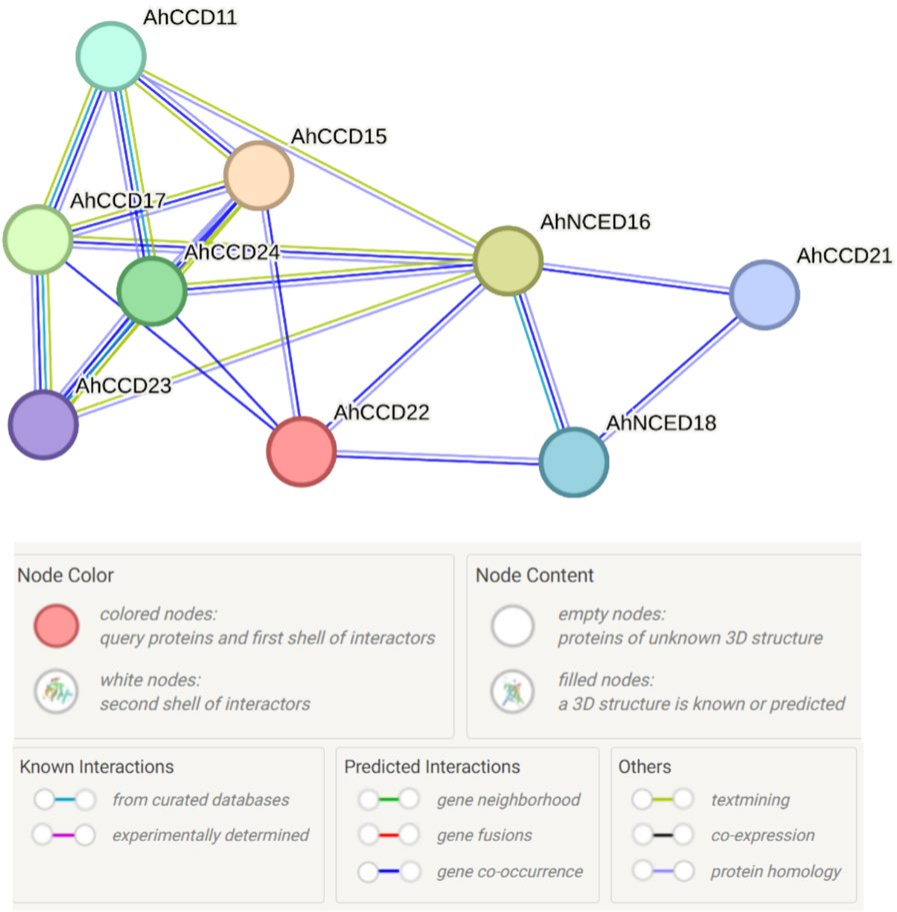
Protein interaction figure depicted the predicted and known interactions among the identified *AhCCO* genes. Most of the *AhCCO* genes make interaction to identified genes *AhCCD24, AhCCD17*, and *AhNCED16* shown in Fig. 7

Only nine genes (out of 24) show linkage that describes the tight interaction with their molecular structure in protein-protein interaction structure analysis. The genes *AhCCD24*, *AhCCD17*, and *AhNCED16 *have the most linkage (Fig. 7), whereas *AhCCD22 *and *AhCCD21 *have the least, according to the STRING database [30]. These genes are involved in a variety of roles, including biological processes and molecular functions.

### Chromosomal localization of *AhCCO* genes

**Fig. 8:**
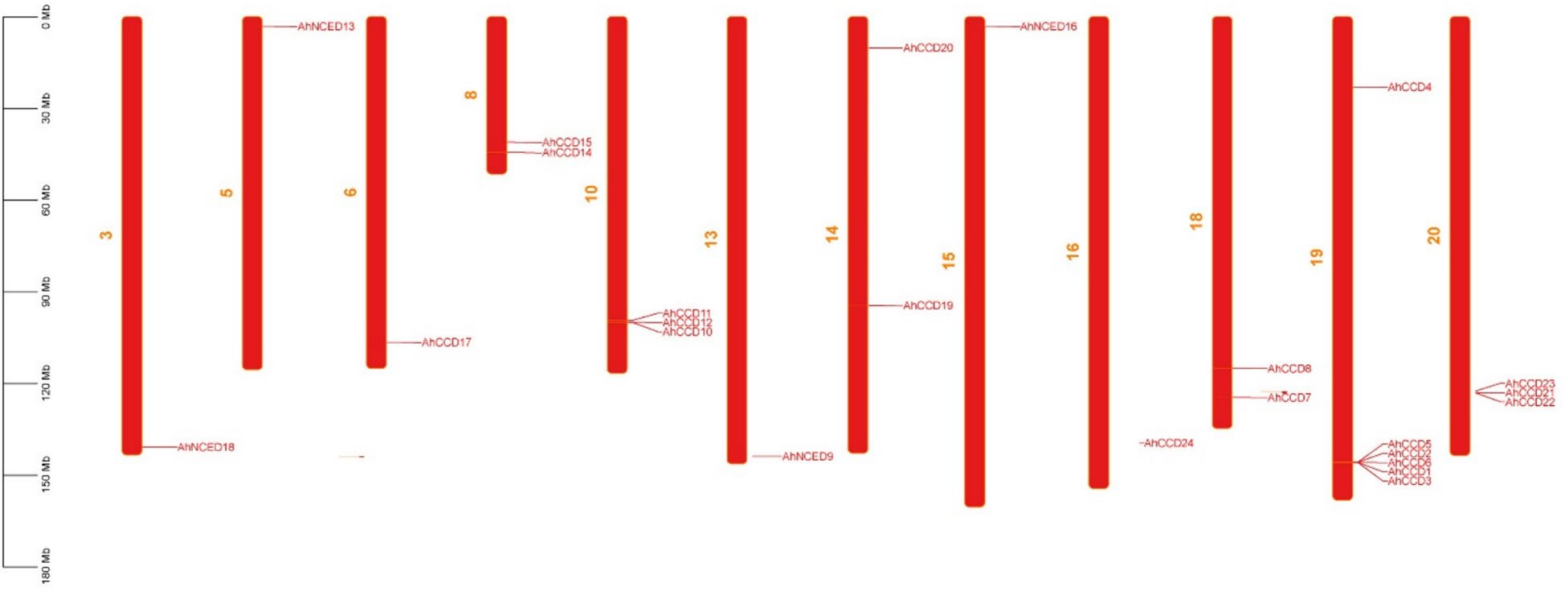
Chromosomal mapping shows the paralogues of *CCO* genes with plausible locations. Genes are duplicated during selection pressure and genomic rearrangement, resulting in the retention of ancestral function and the gain of stable functional characteristics in the peanut genome

There are a total of 12 chromosomes found, containing 24 peanut CCO genes spread on different chromosomal numbers (Fig. 8). Chromosome 19 has the most genes (6 genes; 25%), chromosome 10 and 20 have 3 (12.5%), chromosome 18, 8, 14 have 2 (8.3%), and chromosome 13, 15, 5, 6, 3 and 16 have only single genes (4.16%).

**Fig. 9:**
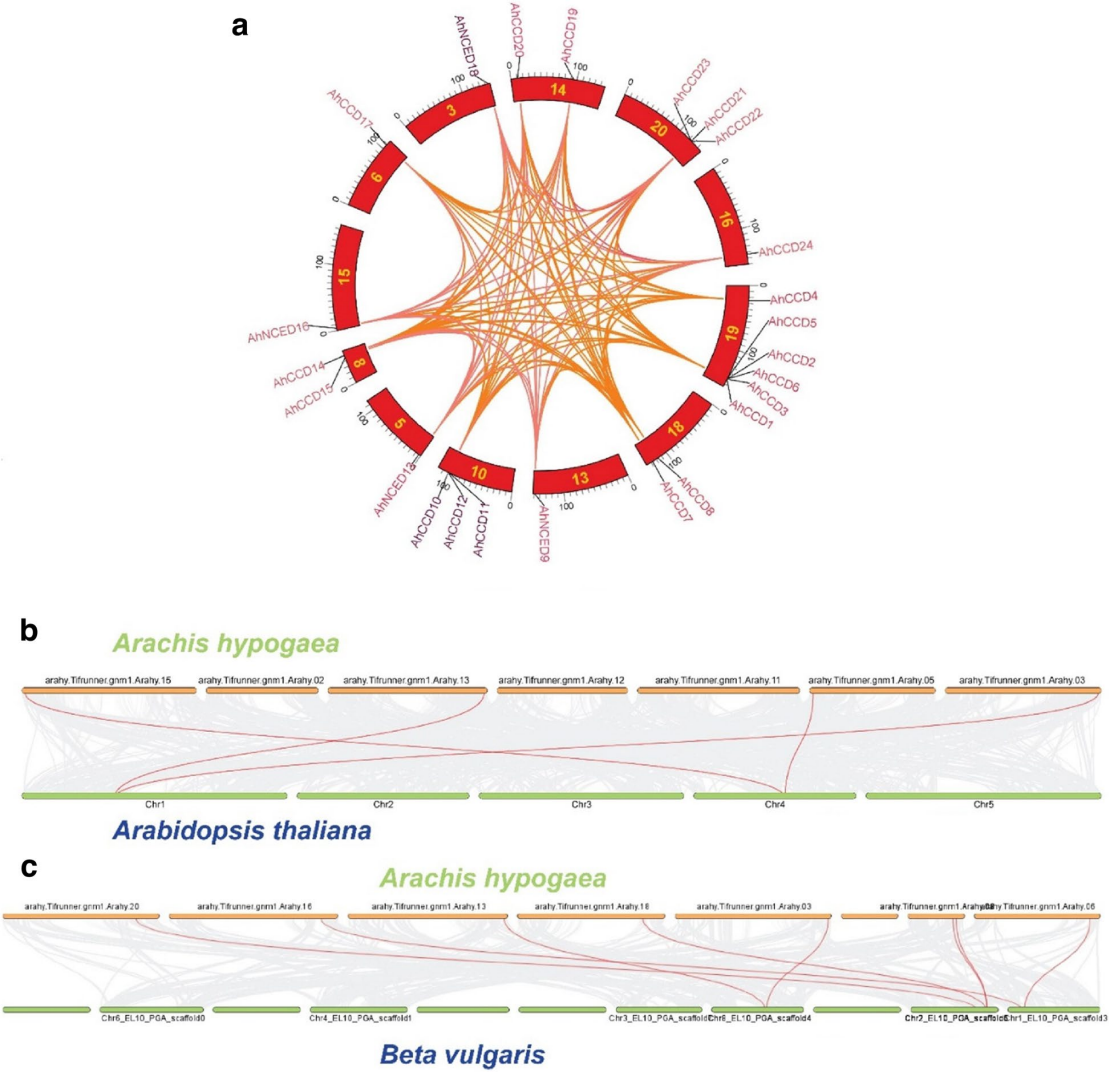
To reveal the similarity and gene duplication distribution, single (**a**) and additional dual synteny analyses of *A. hypogaea* were conducted. *Arachis-Arabidopsis *(**b**), *Arachis-Beta *(**c**) depicted the linkage gene duplication of both specimens. The genomes mentioned plant species have been found to share minor structural and gene duplication sharing

Additional analysis of synteny (depicted in Figs. 9a, b, and c) revealed the genetic linkage between chromosome 1 of *A. thaliana* and *A. hypogaea* genes (*AhNCED9*, *AhNCED18*), and chromosome 2 of *A. thaliana* with genes (*AhNCED13*, *AhNCED16*). Furthermore, the linkage was observed between chromosomes 1, 2, and 8 of *B. vulgaris* with *A. hypogaea* genes (*AhNCED9*, *AhNCED18*), genes (*AhCCD14*, *AhCCD15, AhCCD8, AhCCD11, AhCCD23*), and genes (*AhCCD17*, *AhCCD24*) respectively.

### Gene expression analysis of different *A. hypogaea* organ

**Fig. 10:**
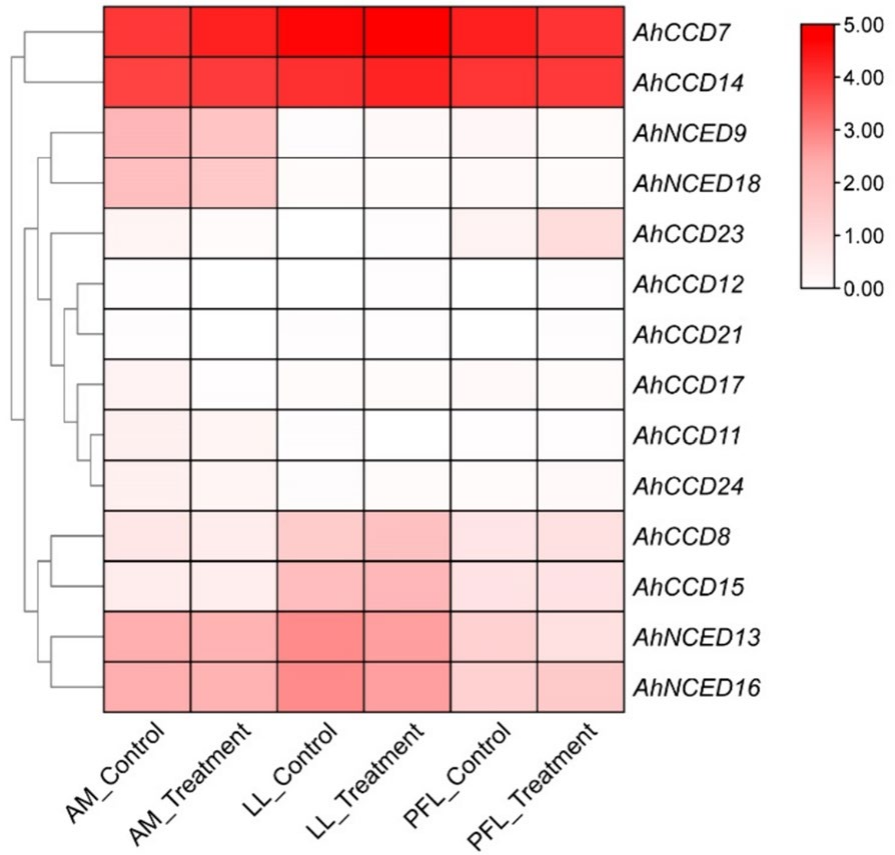
Heatmap show the development of compound Lateral leaf (LL), Apical Meristem (AM) and Pentafoliate Leaf (PFL) Triggered by up-regulatory genes shown. AM Control, LL Control and PFL Control were the Leaves of Mutant cultivar of peanut plant while AM Treatment, LL Treatment and PFL Treatment were the wild type

A brief study of the *CCO *gene family in the context of *A. hypogaea* (peanut) research has resulted in the discovery of 24 genes. Four of these genes (*AhCCD7, AhCCD14, AhCCD23, and AhCCD17*) have been found to be up-regulated in the apical meristem (AM) (Fig. 10). The gene *AhCCD14 *is up-regulated during lateral leaf (LL) development; however, another two genes (*AhCCD12 and AhCCD21*) are up-regulated during pentafoliate leaf (PFL) development. Significantly, one gene from the apical meristem, two from the lateral leaf, and one from the pentafoliate leaf development stages indicate down-regulatory patterns. This study shows the complex regulatory role of these genes in different phases of *A. hypogaea* growth, stress responses, the nutrient cycle and gas exchange mechanisms also in development.

### Gene expression analysis of biotic stress on *A. hypogaea* plant parts and resistance

**Fig. 11:**
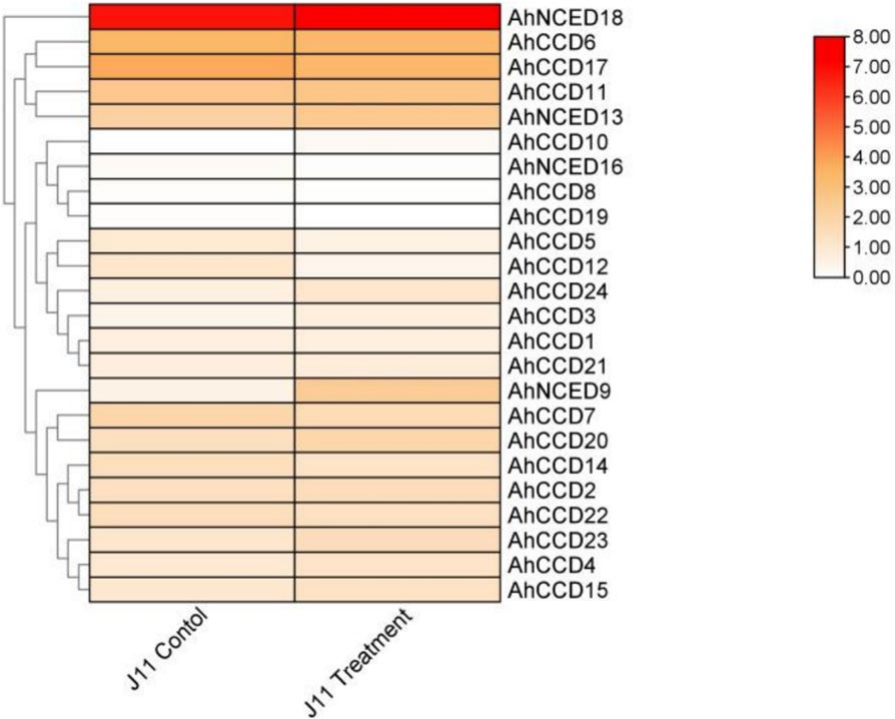
Heatmap shows the resistance of the pathogen fungus (*A. flavus*) of peanut plants to their pre-harvest stage that is triggered by an up-regulated gene in red as the maximum

The observed response of two distinct genes out of 24, *AhNCED9 *and *AhNCED13*, both of which exhibit considerable up-regulation, is especially important to our investigation (Fig. 11). This elevated expression profile is seen in the context of peanut pre-harvest seeds that demonstrated resistance to the fungus *A. flavus* [31]. A single gene in the investigated retrieval seems to be down-regulated, increasing the variety of regulatory responses. This in-depth assessment reveals the genetic complexity behind the peanut plant’s resistance mechanisms in combating the effects of the fungal disease, providing a description of potential approaches to enhancing peanut crop resilience and productivity in the presence of *A. flavus*.

## Discussion

Using bioinformatics tools, an enormous number of *CCOs *genes have been found and analyzed in many species in recent years. Advancements in the *AhCCO *peanut family’s research and development have lagged behind those seen in other species. There is currently inadequate information available on *CCOs *in *A. hypogaea*. The major mechanisms driving species evolution, producing novel gene functions, and fostering the creation of gene families are gene loss and duplication. Members of the *CCO *gene family have been found in a variety of plants. Gene duplication indicates the diversifying or purifying selection based on ka/ks value. Four *AhCCD *paralogous-gene pairs show diversifying selection because of ka/ks values greater than 1 among all. This categorization further aids in discerning gene functions based on their respective categories. As a result, phylogenetic analysis could play a role in the development of functional genomics. The phylogenetic analysis predicts the gene categorization of *CCD *and *NCED *in different crops like *A. hypogaea, B. vulgaris*, and *A. thaliana* and also shows the genes that are involved in pigmentation and stress response. *A. hypogaea, B. vulgaris*, and *A. thaliana* show four (16%), six (66%), and four (57%), respectively, of *NCED *genes in a total of twenty-four, nine, and seven *CCO* genes in crops. The *NCED *subfamily likely diverged after dicots and monocots split, while the *CCD *subfamily diverged before angiosperms split. The phenomenon of *CCO* gene variances could be the result of gene duplication in various crops. The intron-exon structure analysis (Fig. 6) shows the genes that have a range of intron-exons 6-7 to 14–13, except for two *AhNCED *genes (*AhNCED9 *and *AhNCED18*) that are intronless. This intron-deficient structure used to be believed to be critical for plants’ quick response to stress via ABA production and ABA-mediated signal transduction [32]. In general, most *AhCCO *genes contain two exons. In the present investigation, a substantial proportion of *AhCCO *genes, specifically 20 genes (83.3%), exhibited cytoplasmic abundance, while 12 genes (50%) were identified within the chloroplast, and 11 genes (45.8%) were located within the mitochondria. Notably, the genes detected within the chloroplasts are likely implicated in chlorophyll synthesis via the photosynthesis pathway.

The conserved motif domain-RPE65 expresses the motif compositions and gene structures that are widely distributed in 24 *AhCCO *genes and exhibit the range of motif from 6 to 17, except that 2 (8.33%) *AhCCD* genes express exclusively two motifs and eight (33.3%) *AhCCD* genes express three motifs. Previous research has shown that *CCO *genes play essential roles in ABA regulation and plant stress response [20, 33]. The 24 *AhCCO *genes were found on all 12 chromosomes; however, chromosome 19 has the most genes (25%), while the others have less than 12%. The *CCO *promoters also contained a variety of stress-induced cis-acting elements, including ARE, LTR, MBS, TC-rich repeats, DRE, and GC-motifs. However, most of the *AhCCO *promoters contained an ABRE cis-element, which has significance in ABA responsiveness [34, 35].

Plant reactions to biotic and abiotic stress have been discovered to be triggered by miRNAs [36, 37]. Several miRNA families were predicted to regulate the expression of *CCO *genes in peanut, but ahy-miR394 miRNA that plays a role in regulating plant defense responses to abiotic stress revealed in our research [26]. It was predicted that ahy-miR394 targets the *CCO *gene (*AhCCD15*), which suggests that it could be involved in abiotic stress regulation. Other miRNA may have a role in ABA regulation and several stress responses. Gene expression patterns are frequently associated with gene function. The transcriptomic analysis reveals the multifaceted influence of distinct *CCD *and *NCED *genes on the biotic stress response, growth, and development of *A. hypogaea*. These findings provide insights into the diverse contributions of *CCD *(*AhCCD12, AhCCD21*)genes to the development of various plant parts such as the lateral (LL), apical meristem (AM), and pentafoliate leaf (PFL), as well as their involvement in abiotic stress responses. Conversely, the *NCED *genes (*AhNCED9 *and *AhNCED13)* appear to be particularly associated with pathogenic fungal resistance, representing a distinct and specialized role in the plant's defense mechanisms. These genes help the peanut plant regulate the nutrient cycle and growth of compound lateral and pentafoliate leaf development.

## Conclusion

This is the most comprehensive study of the *AhCCO *gene family in terms of evolutionary relationships, gene structures, conserved motifs, cis-acting areas, and gene expression patterns in *A. hypogaea*. The study of these *AhCCO *genes reveals their evolutionary relationship to other *CCO *genes from different crops and helps to find out their closely related functions. Furthermore, an extensive study of cis-acting elements inside *CCO *gene promoters revealed an abundance of cis-acting elements involved with important functions such as growth, development, phytohormone response, and stress response. The finding precisely suggests that the *CCO *gene family could be involved with a variety of regulatory and transcriptional processes. Subcellular localization predictions demonstrated that a significant amount of *AhCCO *proteins are functionally located inside the cytoplasm and chloroplast. Particularly, the gene expression study revealed that *AhCCD7 *and *AhCCD14 *were overexpressed during the early stages of the development of compound leaves, whereas *AhNCED18 *was over-expressed in response to biotic stresses such as pathogenic fungal resistance. The findings of this research hold important significance for improving our understanding of the genetic complexities underlying *AhCCO *genes. Furthermore, these results provide potential for encouraging the development of more robust *A. hypogaea* cultivars capable of withstanding both growth barriers and biotic stresses, thus contributing to the long-term improvement of peanut farming.

## Data Availability

All data generated or analyzed during this study are included in this published article.
